# Bladder Malakoplakia in Systemic Sclerosis Patient: A Case Report and Review Literature

**DOI:** 10.1089/cren.2018.0038

**Published:** 2018-06-01

**Authors:** Wichien Sirithanaphol, Sakkarn Sangkhamanon, Sittichai Netwijitpan, Chingching Foocharoen

**Affiliations:** ^1^ Division of Urology, Department of Surgery, Faculty of Medicine, Khon Kaen University, Khon Kaen, Thailand.; ^2^ Pathology Department, Faculty of Medicine, Khon Kaen University, Khon Kaen, Thailand.; ^3^ Division of Allergy-Immunology-Rheumatology, Faculty of Medicine, Khon Kaen University, Khon Kaen, Thailand.

**Keywords:** malakoplakia, systemic sclerosis, granulomatous disease, scleroderma, hematuria

## Abstract

Malakoplakia, an anecdotal reactive granulomatous lesion, is a rare pathologic entity but relatively more common in genitourinary tracts. Here we report a case of malakoplakia in the urinary bladder in systemic sclerosis. The patient was a 66-year-old female treated with long-term corticosteroid and cyclophosphamide. She presented with gross hematuria, and cystoscopy and biopsy revealed malakoplakia. There was no tumor and the stains for infectious organism were all negative. To the best of our knowledge, this is the first case report of malakoplakia in a systemic sclerosis patient.

## Introduction and Background

Systemic sclerosis is an uncommon connective tissue disease involving skin and vessels, thus affecting all systems of the body. Patients usually present with thickening of the skin and further develop internal organ involvement, for example, pulmonary fibrosis, pulmonary hypertension, or renal failure. Treatment usually includes immunosuppressive agents.

Malakoplakia, the rare granulomatous condition, affects all organs but predominantly the genitourinary tract system. Although the pathogenesis is uncertain, it is thought to develop from chronic infection especially in immunocompromized patients related to defective activity of macrophages.^1^ It is recently described in renal transplant recipients and cancer patients receiving chemotherapy.^2–4^ We report a first case of malakoplakia in a systemic sclerosis patient.

## Case Report

A 66-year-old woman presented with avascular necrosis in left hip, panniculitis, and later was found with diffuse systemic sclerosis and pulmonary fibrosis. She received low-dose methotrexate (15 mg/week) combined with prednisolone 5 mg b.i.d since 2014. Cyclophosphamide 50 mg twice daily was added in January 2015 and then further reduced to once daily in July 2016. The disease was well controlled with no further visceral organ involvement.

Two years later, she complained of gross hematuria and dysuria. She reported no fever, no suprapubic or flank pain, and no loss of appetite. Clinical examination revealed no abnormality except the diffuse skin thickening from her underlying disease. Urinalysis showed >100 red blood cells and leukocytes/μL, whereas urine culture was negative for organism. Her kidney function was within normal limit. Ultrasonography of the kidney, ureter, and bladder system revealed normal kidneys and turbid urine in the urinary bladder. No vesical mass or stone was detected.

**FIG. 1.**
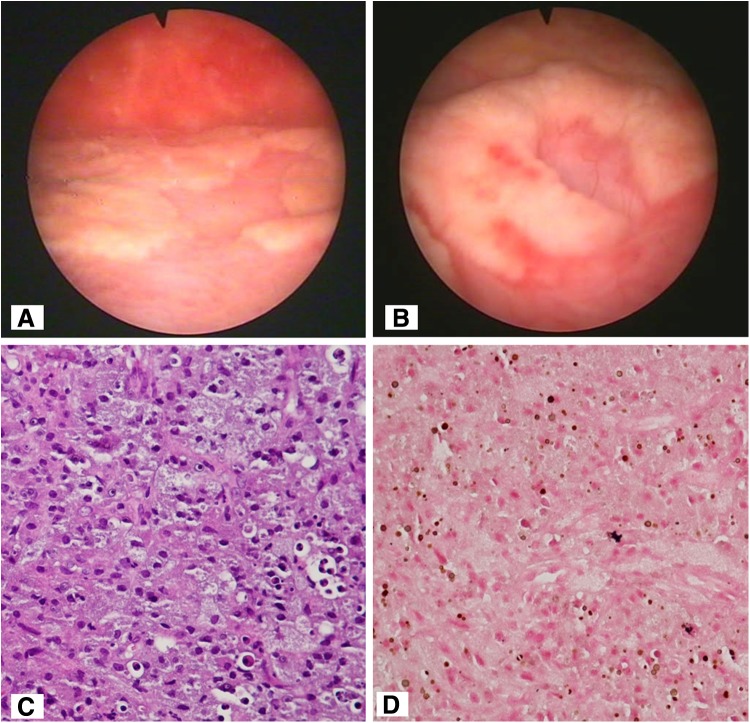
**(A, B)** A white-yellowish plaque in the bladder by cystoscopy. **(C)** Sheets of large macrophages with granular eosinophilic cytoplasm and mixed inflammatory cell infiltration using heamatoxylin and eosin staining (magnification: 400×). **(D)** Michaelis-Gutmann bodies using von Kossa staining (400×).

Cystoscopy was done and showed a white-yellowish plaque in the bladder (Fig. 1A, B). Hematoxylin and eosin stain (magnification: 400 × ) shows sheets of large macrophages with granular eosinophilic cytoplasm and mixed inflammatory cells infiltration (Fig. 1C). Stain of von Kossa (400 × ) shows Michaelis–Gutmann bodies (Fig. 1D).

The cyclophosphamide was permanently stopped, and she was treated with ciprofloxacin 500 mg orally twice daily for 2 weeks. She did not receive any intravenous antibiotics. She reported neither hematuria nor dysuria after full course of antibiotics and the urinalysis showed 5–10 leukocytes/μL and 1–2 red blood cells/μL. She continued the prophylactic dose of trimethoprim–sulfamethoxazole as well as prednisolone and methotrexate.

## Discussion

Malakoplakia is an uncommon granulomatous disease that affects mainly the genitourinary tract. It has been described mostly in immunocompromized conditions such as, solid organ transplantation, human immunodeficiency virus infection, diabetes, malignant tumors, and autoimmune diseases. Although the exact pathogenesis is not known, it is hypothesized that malakoplakia results from macrophage function defect in response to chronic bacterial infection.

Patients with malakoplakia of the bladder present with irritative symptoms or hematuria. The cystoscopic findings include plaques, nodules, and masses that resemble tumors. The diagnosis is made by biopsy. The histology analysis is pathognomonic for the condition; the lesion is characterized by aggregates of histiocytes (von Hansemann cells) containing granules with deposits of iron and calcium called Michaelis–Gutman bodies.

Urinary symptoms are frequent in systemic sclerosis. Mobility limitation because of joint stiffness and skin thickening, myopathy, forced diuresis because of cardiac and pulmonary involvement, as well as medications put patients at higher risk of voiding dysfunction. Furthermore, urinary tract infection is common because of immunosuppressive drugs and bladder filling dysfunction.

We performed a literature search with PubMed, Scopus, and Web of Science; there was no article reporting case of malakoplakia in a scleroderma patient. This is the first case report of this rare condition in systemic sclerosis. Physicians treating scleroderma patients with lower urinary tract symptoms or hematuria should be vigilant if the condition does not resolve after treatment. Although it is uncommon, it is important to maintain a suspicion for malakoplakia, as this is the key to diagnosis and treatment. Further studies in large-scale scleroderma patients are warranted.

## References

[1] Abdou NI, NaPombejara C, Sagawa A, et al. Malakoplakia: evidence for monocyte lysosomal abnormality correctable by cholinergic agonist in vitro and in vivo. N Engl J Med 1977;297:1413–1419 200843 10.1056/NEJM197712292972601

[2] Mohacsi G, Julesz J, Berger Z, Ormos J Bilateral renal malacoplakia in systemic lupus erythematosus and adrenogenital syndrome. Int Urol Nephrol 1989;21:31–38 2714948 10.1007/BF02549899

[3] Nieto-Rios JF, Ramirez I, Zuluaga-Quintero M, Serna-Higuita LM, Gaviria-Gil F, Velez-Hoyos A Malakoplakia after kidney transplantation: Case report and literature review. Transpl Infect Dis 2017;19 10.1111/tid.12731 28561517

[4] Sachanas S, Pangalis GA, Karouzakis P, Koulieris E, Moschogiannis M, Kalpadakis C, Yiakoumis X, Rontogianni D Malakoplakia of the urinary bladder in a patient with chronic lymphocytic leukemia under ibrutinib therapy: A case report. Anticancer Res 2016;36:4759–4762 27630324 10.21873/anticanres.11032

